# Supplementary material for: Transendothelial migration of the Lyme disease spirochete involves spirochete internalization as an intermediate step through a transcellular pathway that involves Cdc42 and Rac1
Source: Microbiol Spectr. 2024 Dec 27;13(2):e02221-24. doi: 10.1128/spectrum.02221-24 (PMC11792520; doi:10.1128/spectrum.02221-24)
Supplement: Supplemental tables and figures — Tables S1 to S3; Fig. S1 to S6. [file spectrum.02221-24-s0001.pdf]

Supplementary Material - Alvarez-Olmedo, Kamaliddin, Verhey, Ho, DeVinney and Chaconas

Transendothelial migration of the Lyme disease spirochete involves spirochete internalization as an intermediate step through a transcellular pathway that involves Cdc42 and Rac1

Published studies – HUVEC cells

Reference	*Borrelia*/Media	Co-culture time	Co-culture medium
(1)	BSK-II	4h	M199 + 20% FBS
(2)	BSK-II 6% RS	16h	M199 + 20% HiFBS
(3)	BSK-II	6 to 72h	DMEM
(4)	MKP Human serum	3h, 6h, 24h	Cell medium without antibiotic

Published studies – Primary cells/cell lines

Reference	*Borrelia* culture media	Co-culture time	Co-culture medium
(5)	BSK-II + 6% RS	2 to 18h	DMEM/M199 +10% FBS
(6)	BSK-II	Up to 3d	No detailed
(7)	Serum free BSK	4h/8h/12h/24h	M199 + 20% HiFBS
(8)	BSK	8h	M199 + 20% HiFBS
(9)	BSK	4h	M199 + 20% HiFBS
(10)	Serum free BSK	5h	M199 + 20% HiFBS + HEPES
(11)	Serum free BSK	1h	M199
(12)	BSK II 10% RS		M199 10% HiFBS
(13)	BSK-II 6% RS	24h	RPMI or RPMI 10% FBS and 20% BSK-II medium (RPMI-B)
(14)	BSK-II 6% RS	Over night	RPMI-B

Table S1. Published media suitable for short-term co-culture of *B. burgdorferi* and HUVEC or primary cells.

RS = rabbit serum

FBS = fetal bovine serum

HiFBS = heat inactivated fetal bovine serum

GCB strain	Description	Missing plasmids	Source/ Reference
705	B31-A (15) + pTM61 *gent,gfp.* It is missing the following plasmids: lp21, lp25, lp28-1, lp28-4, lp36, cp9, cp32-6 and cp32-8.		(16)
726	B31 5A4 NP1 (*kan*) (17) + pTM61 *gent,gfp*		(16)
847	B31-A3 (18) + pTM61 *gent,gfp,* clone 23		(19)
849	B31-A3 (18) *Δp66:kan* (K04 C3-14) + pTM61-*strep,gfp*, clone 1		(19)
3003	B31-A3 (18) K04 C3-14 + *p66*^{D205A,D207A} *gent,* restored to chromosome clone 2-30 + pTM61-*strep,gfp*, clone 2-1		(19)
3007	B31-A3 (18) *ΔospC::kan* + pTM61-*strep*		(20)
4032	B31 5A4 (21) *ΔdbpA,B::gent,* pTM61-*kan,gfp*	cp9, lp21	(22)
4080	B31-5A17 (21)*, +* pTM61-*kan,gfp,pncA,vlsE*$_{A3}$	lp25, lp28	(23)
4036	5A17 (21) *Δbbk32::strep* + pTM61-*kan,gfp,pncA*	lp25, lp28, cp9	(23)
4043	5A17 (21) + pTM61-*kan,gfp,pncA*	lp25, lp28, cp9	(23)
4446	B31 5A4 pTM61*kan,gfp*		(22)
4452	B31-A3 (18) *ΔospC::kan* + pTM61*gent,gfp,ospC*$_{B31\text{-}ECM}$	cp9	(20)
4458	B31-A3 (18) Δ*ospC::kan* + pTM61*gent,gfp,ospC*$_{B31}$	lp28-4, lp56, cp9	(20)
4517	5A17 (21) *Δbbk32*::*strep* + pTM61*kan*,*gfp*,*pncA*,*vlsE*$_{A3}$	lp25, lp28, cp9	(23)

Table S2. Bacterial strains used in this work.

INHIBITOR	TARGET/PATHWAY	DOSES TESTED	TRANSMIGRATION	METABOLIC ACTIVITY IN CELLS	PRESENCE OF MONOLAYER INTEGRITY	% CELL INFECTED	*B. burgdorferi* LOAD
**LY294002**	PI3K (24).	10-40 μM	Apparent increase*	Decreased	No	NA	NA
**U0126**	MEK1/2	10-30 μM	Apparent increase*	Decreased	No	NA	NA
**Cilengitide**	Integrins (25).	1-10 μM	Apparent increase*	NA	No	NA	NA
**Filipin III**	Flotillin-dependent Caveolin-dependent	0.83-5 μM	Apparent increase*	NA	Yes	NA	NA
**Dynasore**	Dynamin inhibitor of GTPase activity of dynamin 1/2 (it inhibits dynamine mediated endocytosis) (26).	80 μM	No change	NA	NA	NA	NA
**Dasatinib**	Src, Abl (27).	1-20 nM	No change	NA	NA	NA	NA
**CK-666**	Arp2/3 complex cell-permeable inhibitor (28).	4-30 μM	No change	Decreased	Yes	NA	NA
**Imipramine**	EGFR/PKC-δ/NF-κB signaling. Macropinocytosis inhibitor (29).	5-40 μM	No change	No changed up to 5 μM; decreased at higher doses	Yes	NA	NA
**SB203580**	P38 inhibitor	10-40 μM	No change	Decreased	NA	NA	NA
**amiloride**	Macropinocytosis inhibitor (inhibits recruitment of Cdc42/Rac1 to the membrane (30).	6-7.5 μM	DECREASED	No changed	Yes	DECREASED	DECREASED
**ML141**	Cdc42 selective allosteric, reversible inhibitor. At dose higher than 100 μM, it can inhibit other Rho family GTPases such as Rac1 (31).	10-30 μM	DECREASED	No changed	Yes	DECREASED	DECREASED
**EHop-016**	Rac1/3 until 5 μM; Rac1/3 and partial inhibition of Cdc42 at higher dose (32).	0.1-10 μM	DECREASED at 5 μM No change when the monolayer integrity was lost (Bb can probably use a paracellular route in this case).	Decreased	Yes, up to 5 μM. Integrity lost with higher dose (10 μM).	INCREASED	INCREASED
**NSC 23766**	Inhibitor of Rac1 activation by Rac-GEFs.1 (33).	15-50 μM	DECREASED	No changed	Yes	No change	INCREASED

Table S3. Inhibitors tested in this manuscript: Effect on *B. burgdorferi* transendothelial migration in Transwells.

Note: (NA) not assayed. Highlighted areas indicate relevant changes.

*Apparent increase: may be due to monolayer integrity loss.

#Median fluorescence intensity per cell (see **Figs. 8-10**).

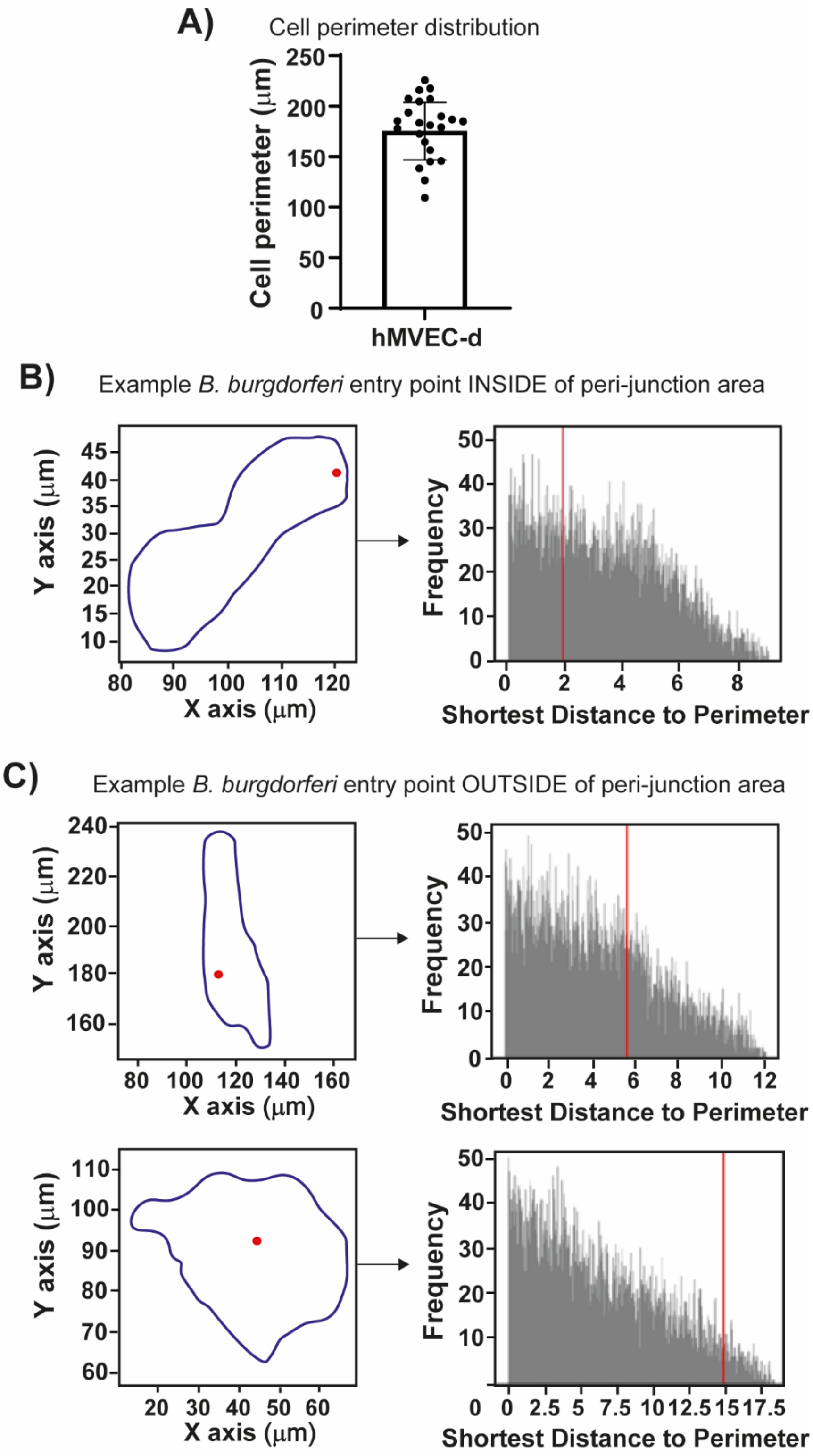

Fig. S1. Examples of cell shape and *B. burgdorferi* entry point distribution

A) Estimation of the size of the hMVEC-d cells: the perimeter of 30 cells were measured in Fiji, the average of the perimeter was 175.23 um +/- 28.36. **B)** Example of *B. burgdorferi* entry point distribution inside what was considered the peri-junction area: the micrographs showing penetrating spirochetes were used to identify coordinates of the perimeter (blue) and penetration point (red). The perimeter

was used to generate randomly distributed points from a continuous uniform distribution (righ panel). Finally, the shortest distance to the perimeter was computed for the penetration point (red) and the null distribution (grey). **C)** Examples of *B. burgdorferi* entry point distribution outside of what was considered the peri-junction area. It can be appreciated the difference in shape between the cells (top and bottom panel).

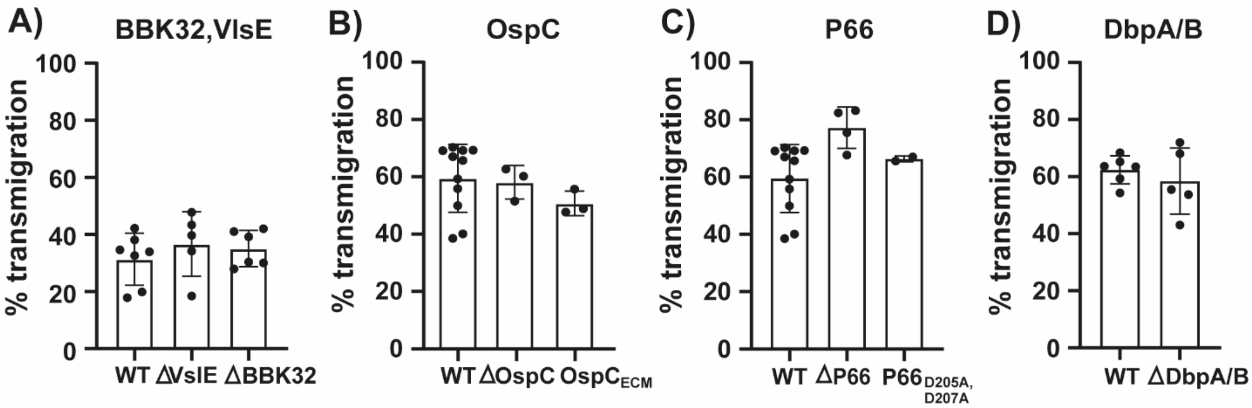

Fig. S2. Assessment of the role of adhesin mutants in *B. burgdorferi* transmigration *in vitro*. A) Evaluation of BBK32 and VlsE: Transwell chambers were seeded with hTERT as described previously, and the upper chamber was infected with $3x10^5$ spirochetes (WT, GCB4080; VlsE knockout, GCB4043; and BBK32 knockout, GCB4017). The percentage of total transmigrated spirochetes was assessed after 20 h as described earlier. Statistics were evaluated using the Kruskal-Wallis test and Dunn's multiple comparison test for BBK32 and VlsE. **B)** Evaluation of OspC requirement for transmigration. Statistics were evaluated using the Kruskal-Wallis test and Dunn's multiple comparison test. **C)** Evaluation of P66 requirement for transmigration. Statistics were evaluated using the Kruskal-Wallis test and Dunn's multiple comparison test. **D)** Evaluation of DbpA/B requirement for transmigration. Statistic was evaluated using Mann Withney test. In all cases, the graphs show the percentage (mean +/- SD) of *B. burgdorferi* traversing human microvascular endothelial cells in three independent experiments with 1-4 samples for each experimental condition .

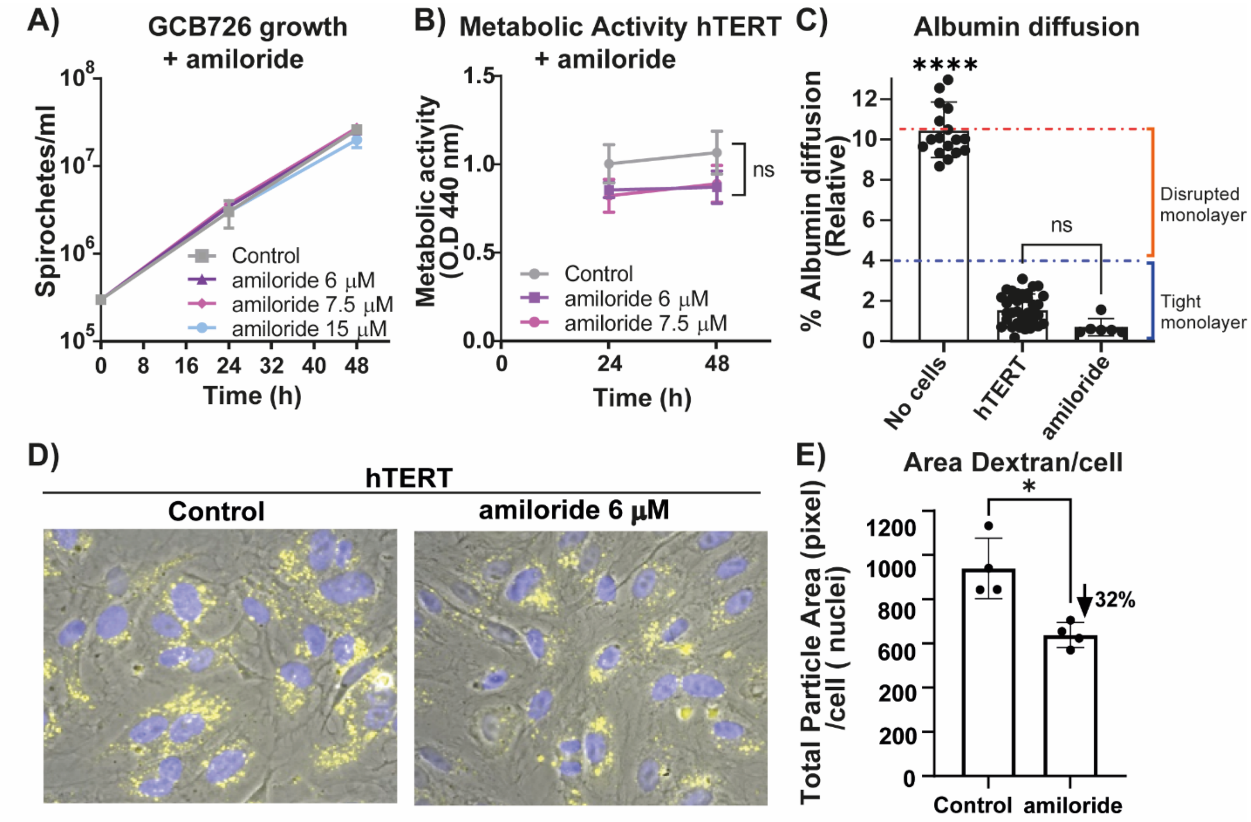

Figure S3: Analysis of amiloride effect in *B. burgdorferi* and hTERT cells.

A) Evaluation of the effect of amiloride on *B. burgdorferi* growth. BSK-II media lacking, or with the indicated concentrations of amiloride, was inoculated to a density of $3x10^5$ Spirochetes/ml. Spirochetes were enumerated every 24 h using a Petroff-Hausser chamber. The graph represents 3 independent experiments, with 1-2 samples for each experimental condition. Statistics were evaluated using mixed-effects analysis with Geisser-Greenhouse correction and Tukey's multiple comparison test and $p < 0.05$ was considered significant, ns = not significant. **B)** The metabolic activity of hTERT cells was evaluated by measuring the optical density (O.D) at 440 nm with the WTS reagent in the presence or absence of the indicated concentrations of amiloride. The cells were seeded in 96 well plates and grown to confluence in VCBM medium, then exposed to amiloride from 24-48 h. The graph represents the results of 3 independent experiments +/-SD (n = 3) normalized to the untreated control at 24 h and analyzed with a two-way ANOVA with Tukey's multiple comparison test; p <0.05 was considered significant, ns = not significant. **C)** Evaluation of the effects of amiloride on monolayer permeability: The assessment of monolayer integrity was performed as indicated previously: 10 µg of Alexa Fluor 555-albumin was added to the upper chamber at 16 h and fluorescence was measure at 20 hours. The graph represents the mean +/- SD of three experiments, with 2-4 samples for each experimental condition. Statistics were evaluated with the Kruskal-Wallis test with Dunn's multiple comparison test. * = $p < 0.05$, **** = $p <$ 0.0001, ns = not significant. **D)** Representative images obtained from an in vitro macropinocytosis assay. hTERT cells were grown on round coverslips to confluence and then treated with fixable-TMR-dextran or amiloride for 1h 30 min in VCBM. After fixing the cells, the nuclei were labeled with DAPI, and the cells mounted on coverslips. A phase contrast image was used to determine the area of the field covered by cells; in all cases, a confluent monolayer was analyzed for the study. Four fields of fluorescent images were captured in the rhodamine channel to show the TMR-dextran-positive macropinocytic puncta. An automatic threshold value was applied to all the images with ImageJ, macropinosomes were detected in red, and once it was accepted, the image was converted to a binary image. In those images, the

macropinosomes were shown in black on a white background (Binary image), watershed processing was performed, and then the resulting image was analyzed with the particle analyzer in ImageJ. **E)** The graph represents the total particle area divided by the number of nuclei on each field in cells maintained in VCBM medium for 1h 30 min. More than 250 cells were analyzed. The data represent the mean +/- SD. Statistical analysis was carried out with Mann-Whitney test,* = $p < 0.05$.

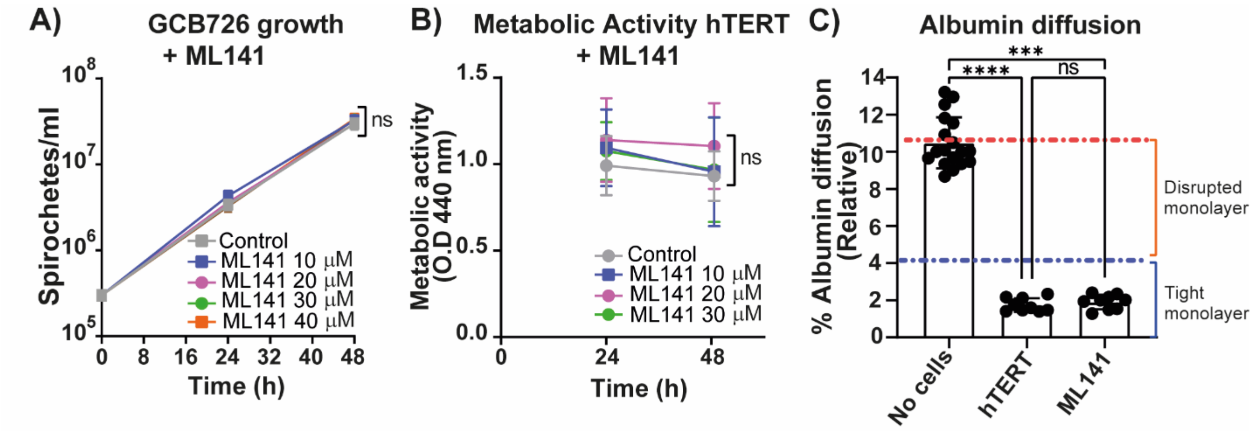

Figure S4: Analysis of ML141 effect in *B. burgdorferi* and hTERT cells.

A) Evaluation of the effect of ML141 on *B. burgdorferi* growth. BSK-II media lacking, or with the indicated concentrations of ML141 (10-40 μM), was inoculated to a density of $3X10^5$ spirochetes/ml. Spirochetes were enumerated every 24 h using a Petroff-Hausser chamber. The graph represents at least 3 independent experiments. Two-way ANOVA with mixed effects with Geisser-Greenhouse correction and Tukey's multiple comparison test. **B)** The metabolic activity of hTERT cells was evaluated by measuring the optical density (O.D) at 440 nm with the WTS reagent in the absence or presence of the indicated concentrations of ML141 (10-30 μM). The cells were seeded in 96 well plates and grown to confluence in VCBM medium, then exposed to ML141 from 24-48 h. The graph represents the results of three independent experiments +/-SD (n = 6) normalized to the untreated control at 24 h and analyzed with a two-way ANOVA with Tukey's multiple comparison test. **C)** Evaluation of the effects of 30 μM ML141 on monolayer permeability. The assessment of monolayer integrity was performed as indicated previously: 10 µg of Alexa Fluor 555-albumin was added to the upper chamber at 16 h and fluorescence was measure at 20 hours. The graph represents the mean +/- SD of three experiments performed in triplicate, measured in duplicate and analyzed with the Kruskal-Wallis test and Dunn's multiple comparison test. ***= $p < 0.001$, **** = $p < 0.0001$, ns = not significant.

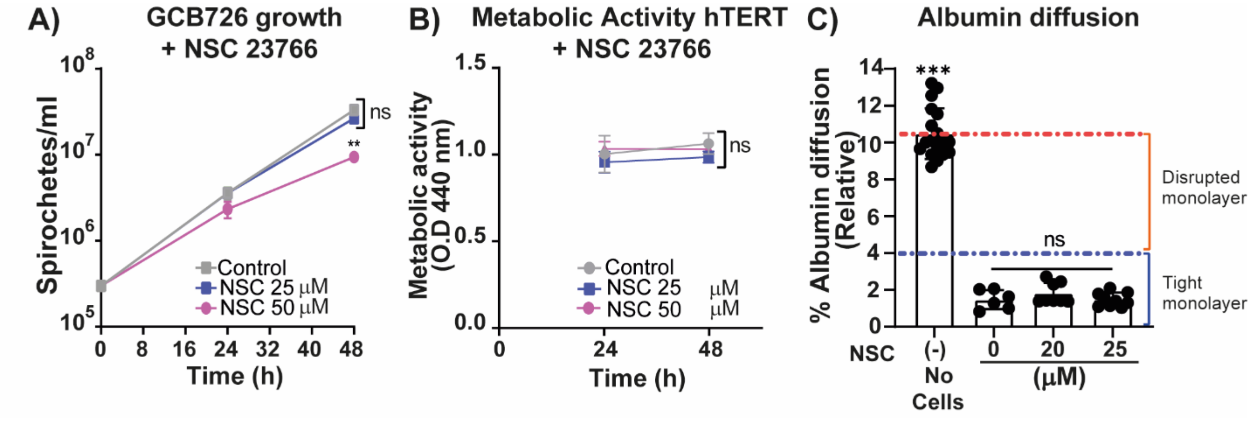

Figure S5: Analysis of NSC 23766 effect in *B. burgdorferi* and hTERT cells.

A) Evaluation of the effect of NSC 23766 on *B. burgdorferi* growth. BSK-II media lacking, or with the indicated concentrations of NSC 23766, was inoculated to a density of $3X10^5$ Spirochetes/ml. Spirochetes were enumerated every 24 h using a Petroff-Hausser chamber. The graph represents at least 3 independent experiments. Two-way repeated measures ANOVA with mixed effects with Geisser-Greenhouse correction and Tukey's multiple comparison test. **B)** The metabolic activity of hTERT cells was evaluated by measuring the optical density (O.D) at 440 nm with the WTS reagent in the presence or absence of the indicated concentrations of NSC 23766 (25-50 µM). The cells were seeded in 96 well plates and grown to confluence in VCBM medium, then exposed to NSC 23766 from 24-48 h. The graph represents the results of 3 independent experiments +/-SD (n = 4) normalized to the untreated control at 24 h and analyzed with with a two-way ANOVA with Tukey's multiple comparison test. **C)** Evaluation of the effects of NSC 23766 on monolayer permeability: The assessment of monolayer integrity was performed as indicated previously: 10 µg of Alexa Fluor 555-albumin was added to the upper chamber at 16 h and fluorescence was measure at 20 hours. The graph represents the mean +/- SD of three experiments performed in triplicate, measured in duplicate and analyzed with the Kruskal-Wallis test and Dunn's multiple comparison. ** = $p < 0.01$, *** = $p < 0.001$, ns = not significant.

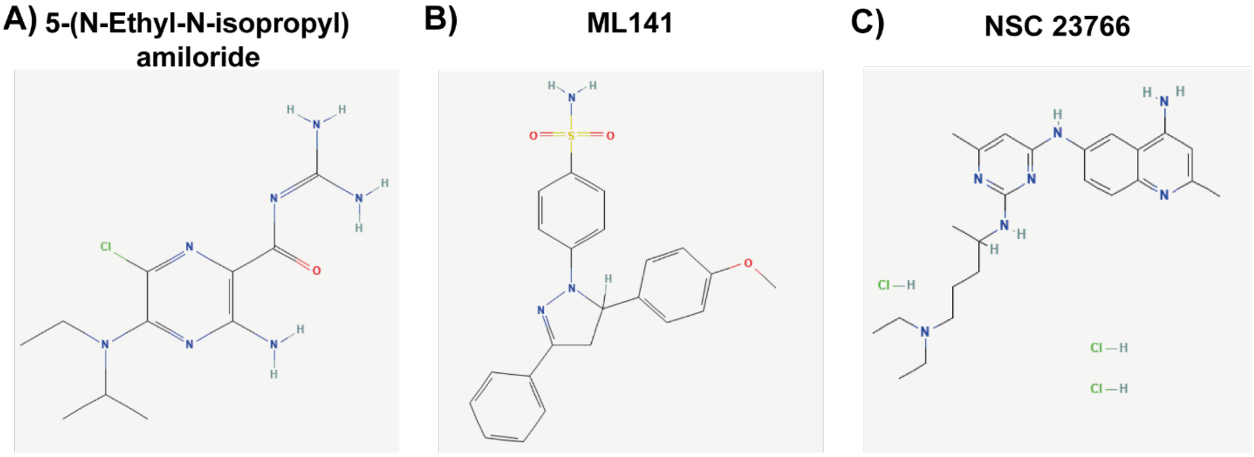

Figure S6: Chemical Structure of the inhibitors used.

A) 5-(N-Ethyl-N-isopropyl)amiloride, used to inhibit the membrane recruitment of Cdc42 and Rac1. **B)** ML141 used to selectively inhibit Cdc42. **C)** NSC 23766 trihydrochloride used to inhibit Rac1. Structures from PubChem.

References

1. Thomas D, Comstock L. 1989. Interaction of Lyme disease spirochetes with cultured eucaryotic cells. Infect Immun 57:1324-26.
2. Grab DJ, Nyarko E, Nikolskaia OV, Kim YV, Dumler JS. 2009. Human brain microvascular endothelial cell traversal by *Borrelia burgdorferi* requires calcium signaling. Clin Microbiol Infect 15:422-6.
3. Brissette CA, Kees ED, Burke MM, Gaultney RA, Floden AM, Watt JA. 2013. The multifaceted responses of primary human astrocytes and brain microvascular endothelial cells to the Lyme disease spirochete, Borrelia burgdorferi. ASN neuro 5:AN20130010.
4. Lafrance ME, Pierce JV, Antonara S, Coburn J. 2011. The *Borrelia burgdorferi* integrin ligand, P66, affects gene expression by human cells in culture. Infect Immun.
5. Wu J, Weening EH, Faske JB, Hook M, Skare JT. 2011. Invasion of eukaryotic cells by *Borrelia burgdorferi* requires β1 integrins and Src kinase activity. Infect Immun 79:1338-1348.
6. Ma Y, Sturrock A, Weis JJ. 1991. Intracellular localization of *Borrelia burgdorferi* within human endothelial cells. Infect Immun 59:671-8.
7. Burns MJ, Sellati TJ, Teng EI, Furie M. 1997. Production of interleukin-8 (IL-8) by cultured endothelial cells in response to *Borrelia burgdorferi* occurs independently of secreted IL-1 and tumor necrosis factor alpha and is required for subsequent transendothelial migration of neutrophils. Infect Immun 65:1217-1222.
8. Burns MJ, Furie MB. 1998. *Borrelia burgdorferi* and interleukin-1 promote the transendothelial migration of monocytes in vitro by different mechanisms. Infect Immun 66:4875-83.
9. Sellati TJ, Abrescia LD, Radolf JD, Furie MB. 1996. Outer surface lipoproteins of *Borrelia burgdorferi* activate vascular endothelium *in vitro*. Infect Immun 64:3180-7.
10. Sellati TJ, Burns MJ, Ficazzola MA, Furie MB. 1995. *Borrelia burgdorferi* upregulates expression of adhesion molecules on endothelial cells and promotes transendothelial migration of neutrophils *in vitro*. Infect Immun 63:4439-47.
11. Coleman JL, Sellati TJ, Testa JE, Kew RR, Furie MB, Benach JL. 1995. *Borrelia burgdorferi* binds plasminogen, resulting in enhanced penetration of endothelial monolayers. Infect Immun 63:2478-84.
12. Grab DJ, Perides G, Dumler JS, Kim KJ, Park J, Kim YV, Nikolskaia O, Choi KS, Stins MF, Kim KS. 2005. Borrelia burgdorferi, host-derived proteases, and the blood-brain barrier. Infect Immun 73:1014-22.
13. Lazarus JJ, Kay MA, McCarter AL, Wooten RM. 2008. Viable Borrelia burgdorferi enhances interleukin-10 production and suppresses activation of murine macrophages. Infection and immunity 76:1153-1162.
14. Chung Y, Zhang N, Wooten RM. 2013. Borrelia burgdorferi elicited-IL-10 suppresses the production of inflammatory mediators, phagocytosis, and expression of co-stimulatory receptors by murine macrophages and/or dendritic cells. PLoS One 8:e84980.
15. Bono JL, Elias AF, Kupko JJ, III, Stevenson B, Tilly K, Rosa P. 2000. Efficient targeted mutagenesis in *Borrelia burgdorferi*. J Bacteriol 182:2445-52.
16. Moriarty TJ, Norman MU, Colarusso P, Bankhead T, Kubes P, Chaconas G. 2008. Real-time high resolution 3D imaging of the lyme disease spirochete adhering to and escaping from the vasculature of a living host. PLoS Pathog 4:e1000090.
17. Kawabata H, Norris SJ, Watanabe H. 2004. BBE02 disruption mutants of *Borrelia burgdorferi* B31 have a highly transformable, infectious phenotype. Infect Immun 72:7147-54.
18. Elias AF, Stewart PE, Grimm D, Caimano MJ, Eggers CH, Tilly K, Bono JL, Akins DR, Radolf JD, Schwan TG, Rosa P. 2002. Clonal Polymorphism of *Borrelia burgdorferi* Strain B31 MI: Implications for Mutagenesis in an Infectious Strain Background. Infect Immun 70:2139-2150.

19. Kumar D, Ristow LC, Shi M, Mukherjee P, Caine JA, Lee WY, Kubes P, Coburn J, Chaconas G. 2015. Intravital Imaging of Vascular Transmigration by the Lyme Spirochete: Requirement for the Integrin Binding Residues of the *B. burgdorferi* P66 Protein. PLoS Pathog 11:e1005333.
20. Lin YP, Tan X, Caine JA, Castellanos M, Chaconas G, Coburn J, Leong JM. 2020. Strain-specific joint invasion and colonization by Lyme disease spirochetes is promoted by outer surface protein C. PLoS Pathog 16:e1008516.
21. Purser JE, Norris SJ. 2000. Correlation between plasmid content and infectivity in *Borrelia burgdorferi*. Proc Natl Acad Sci U S A 97:13865-70.
22. Tan X, Castellanos M, Chaconas G. 2023. Choreography of Lyme Disease Spirochete Adhesins To Promote Vascular Escape. Microbiol Spectr doi:10.1128/spectrum.01254-23:e0125423.
23. Tan X, Lin YP, Pereira MJ, Castellanos M, Hahn BL, Anderson P, Coburn J, Leong JM, Chaconas G. 2022. VlsE, the nexus for antigenic variation of the Lyme disease spirochete, also mediates early bacterial attachment to the host microvasculature under shear force. PLoS Pathog 18:e1010511.
24. Chaussade C, Rewcastle GW, Kendall JD, Denny WA, Cho K, Grønning LM, Chong ML, Anagnostou SH, Jackson SP, Daniele N, Shepherd PR. 2007. Evidence for functional redundancy of class IA PI3K isoforms in insulin signalling. Biochem J 404:449-58.
25. Hariharan S, Gustafson D, Holden S, McConkey D, Davis D, Morrow M, Basche M, Gore L, Zang C, O'Bryant CL, Baron A, Gallemann D, Colevas D, Eckhardt SG. 2007. Assessment of the biological and pharmacological effects of the alpha nu beta3 and alpha nu beta5 integrin receptor antagonist, cilengitide (EMD 121974), in patients with advanced solid tumors. Ann Oncol 18:1400-7.
26. Macia E, Ehrlich M, Massol R, Boucrot E, Brunner C, Kirchhausen T. 2006. Dynasore, a cell-permeable inhibitor of dynamin. Dev Cell 10:839-50.
27. Shah NP, Lee FY, Luo R, Jiang Y, Donker M, Akin C. 2006. Dasatinib (BMS-354825) inhibits KITD816V, an imatinib-resistant activating mutation that triggers neoplastic growth in most patients with systemic mastocytosis. Blood 108:286-91.
28. Nolen BJ, Tomasevic N, Russell A, Pierce DW, Jia Z, McCormick CD, Hartman J, Sakowicz R, Pollard TD. 2009. Characterization of two classes of small molecule inhibitors of Arp2/3 complex. Nature 460:1031-4.
29. Lin HP, Singla B, Ghoshal P, Faulkner JL, Cherian-Shaw M, O'Connor PM, She JX, Belin de Chantemele EJ, Csányi G. 2018. Identification of novel macropinocytosis inhibitors using a rational screen of Food and Drug Administration-approved drugs. Br J Pharmacol 175:3640-3655.
30. Koivusalo M, Welch C, Hayashi H, Scott CC, Kim M, Alexander T, Touret N, Hahn KM, Grinstein S. 2010. Amiloride inhibits macropinocytosis by lowering submembranous pH and preventing Rac1 and Cdc42 signaling. Journal of Cell Biology 188:547-563.
31. Hong L, Kenney SR, Phillips GK, Simpson D, Schroeder CE, Nöth J, Romero E, Swanson S, Waller A, Strouse JJ, Carter M, Chigaev A, Ursu O, Oprea T, Hjelle B, Golden JE, Aubé J, Hudson LG, Buranda T, Sklar LA, Wandinger-Ness A. 2013. Characterization of a Cdc42 protein inhibitor and its use as a molecular probe. J Biol Chem 288:8531-8543.
32. Montalvo-Ortiz BL, Castillo-Pichardo L, Hernández E, Humphries-Bickley T, De La Mota-Peynado A, Cubano LA, Vlaar CP, Dharmawardhane S. 2012. Characterization of EHop-016, novel small molecule inhibitor of Rac GTPase. Journal of Biological Chemistry 287:13228-13238.
33. Gao Y, Dickerson JB, Guo F, Zheng J, Zheng Y. 2004. Rational design and characterization of a Rac GTPase-specific small molecule inhibitor. Proceedings of the National Academy of Sciences 101:7618-7623.
